# Incidental contrast opacification of the appendix secondary to urological intervention

**DOI:** 10.1093/jscr/rjad061

**Published:** 2023-02-23

**Authors:** Samuel Hunn, Joshua Winston, Nicholas Davies

**Affiliations:** 14 Jutland St, New Town, Hobart, Tasmania 7008, Australia; 14 Jutland St, New Town, Hobart, Tasmania 7008, Australia

## Abstract

A 23-year-old otherwise well male, with right ureteric stent *in situ* placed electively for a 9-mm symptomatic pelviureteric junction stone underwent a right ureteropyeloscopy, retrograde pyelogram laser lithotripsy and stent exchange for stone clearance. The procedure was uncomplicated. Following stent removal on day 2, the patient developed acute right lower quadrant pain, which was investigated with non-contrast CT abdomen. The scan demonstrated a contrast-filled vermiform appendix, secondary to vicarious contrast excretion. This case report describes a rare manifestation of vicarious contrast excretion and explains this phenomenon.

## INTRODUCTION

Unexpected findings on abdominal imaging in the workup of acute abdominal pain are common. Manifestations of vicarious contrast excretion are one cause for such findings. This case report describes a urological intervention in a 23-year-old male complicated by acute abdominal pain and a unique manifestation of this phenomenon.

## CASE REPORT

A 23-year-old otherwise well male, with right ureteric stent *in situ* placed electively for a 9-mm symptomatic pelviureteric junction stone underwent a right ureteropyeloscopy, retrograde pyelogram laser lithotripsy and stent exchange for stone clearance. The procedure was uncomplicated, with only small residual fragments of stones and no additional stones visualized on image intensifier. The patient was left with a JJ stent on a string for self-removal two days later as per hospital protocol for patients at low risk for ureteric edema.

**Figure 1:**
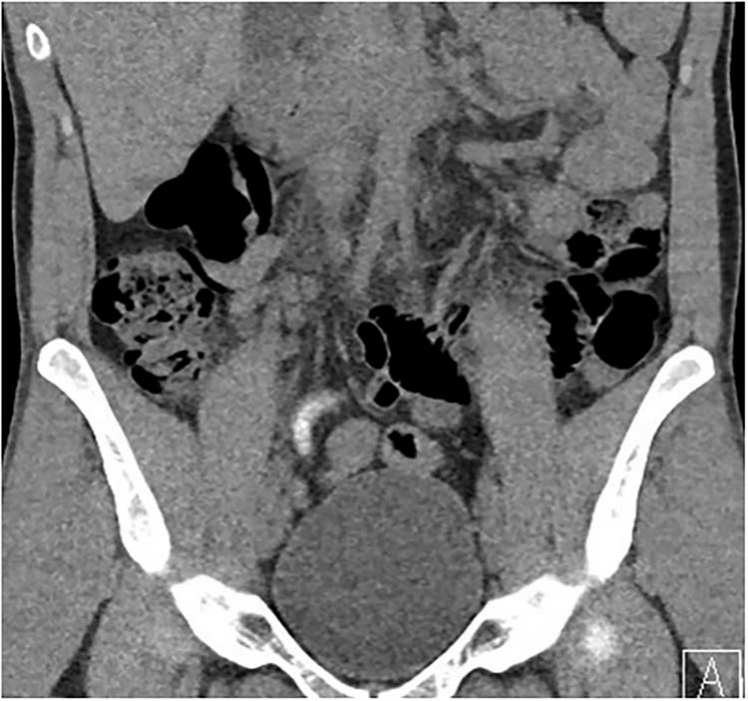
Coronal imaging, demonstrating contrast-filled vermiform structure.

**Figure 2:**
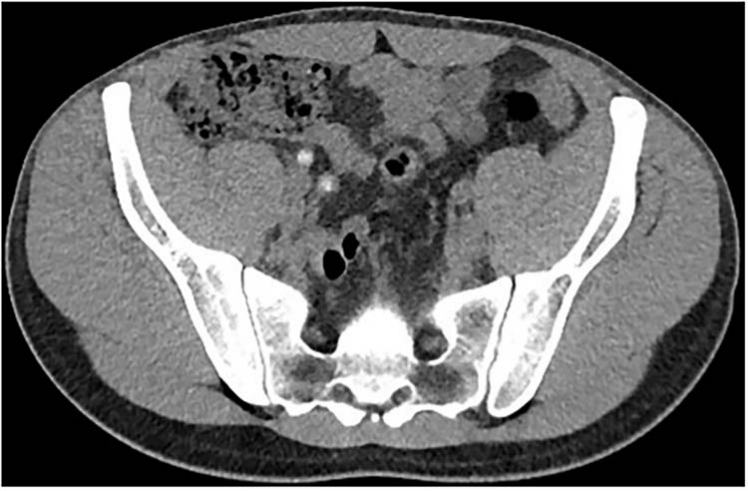
Imaging of the same structure in the axial plane.

**Figure 3:**
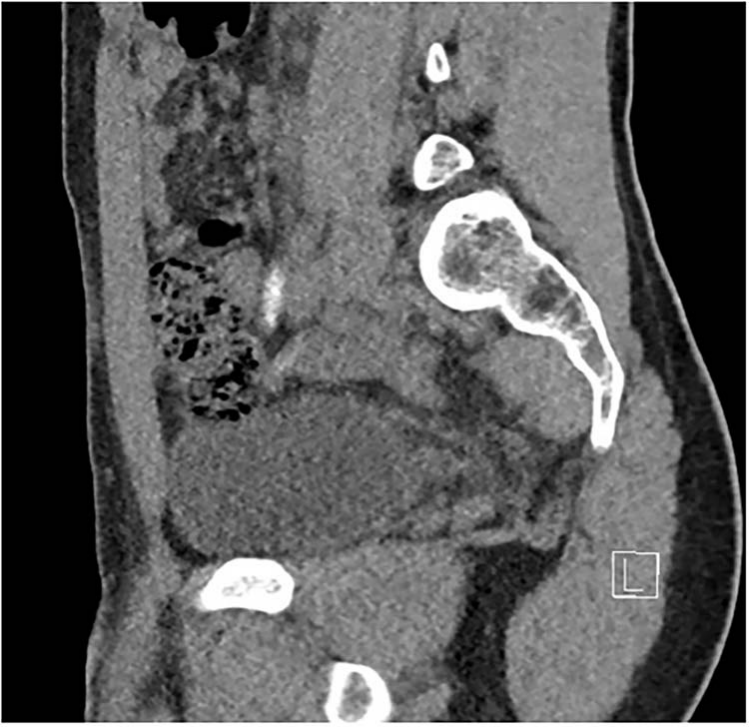
Sagittal imaging, depicting the base of structure arising from the caecum.

Two days following stent removal, the patient developed acute onset right lower quadrant and flank pain. A non-contrast CT of the renal tract was obtained to assess for procedure or stone-related complications. The scan demonstrated two distinct retained stones within the proximal right ureter, with associated moderate ureteronephrosis. Additionally, within the right lower quadrant of the abdomen, a radiopaque vermiform entity was demonstrated (Figs 1–3). This entity was not pathological, however, represented a normal appendix that had, consequent to contrast administration during a urological procedure, become filled with contrast secondary to vicarious contrast excretion.

## DISCUSSION

Vicarious contrast excretion can be seen in instances of obstructive uropathy, or otherwise can be seen in healthy individuals [1, 2]. Vicarious contrast agent is often excreted by the liver, however, may occur via the small bowel [3]. Both scenarios may account for the presence of contrast within the appendix in the presented case. A contrast-filled appendix is generally considered to go against the diagnosis of acute appendicitis, and therefore in a patient complaining of right lower quadrant pain, it can incidentally serve to exclude an alternative cause of pain. However, routine use of oral contrast for the diagnosis of acute appendicitis is thought unnecessary based on available evidence [4]. The given case demonstrates an unusual manifestation of vicarious contrast excretion in a patient with obstructive uropathy and serves as a reminder of how vicarious excretion may manifest in regards to abdominal imaging and the surgical abdomen.
